# The widespread occurrence of tRNA‐derived fragments in *Saccharomyces cerevisiae*

**DOI:** 10.1002/2211-5463.12127

**Published:** 2016-10-21

**Authors:** Kamilla Bąkowska‐Żywicka, Anna M. Mleczko, Marta Kasprzyk, Piotr Machtel, Marek Żywicki, Tomasz Twardowski

**Affiliations:** ^1^ Institute of Bioorganic Chemistry Polish Academy of SciencesPoznanPoland; ^2^ Department of Computational BiologyInstitute of Molecular Biology and BiotechnologyFaculty of Biology A. Mickiewicz University in PoznanPoland

**Keywords:** northern blot hybridization, *Saccharomyces cerevisiae*, small RNAs, tRNA, tRNA‐derived fragments

## Abstract

Short RNAs derived from the cleavage of tRNA molecules are observed in most organisms. Their occurrence seems to be induced by stress conditions, but still little is known about their biogenesis and functions. We find that the recovery of tRNA fragments depends on the RNA isolation method. Using an optimized RNA extraction protocol and northern blot hybridization technique, we show that the tRNA‐derived fragments in yeast are widespread in 12 different growth conditions. We did not observe significant stress‐dependent changes in the amounts of tRNA fragments pool. Instead, we show the differential processing of almost all individual tRNAs. We also provide evidence that 3′‐part‐derived tRNA fragments are as abundant as the 5′‐ one in *Saccharomyces cerevisiae*. The resulting set of *S. cerevisiae* tRNA fragments provides a robust basis for further experimental studies on biological functions of tRFs.

**Abbreviations**
eIF: eukaryotic initiation factor
EXO1: exonuclease 1
GPD1: glycerol‐3‐phosphate dehydrogenase 1
gtRNAdb: genomic tRNA database
HSP12: heat‐shock protein 12
LMW RNA: low molecular weight RNA
PDR12: plasma membrane ATP‐binding cassette (ABC) transporter
*T*_H_: hybridization temperature
*T*_m_: melting temperature
tRF: tRNA‐derived fragment

Transfer RNA are molecules mostly recognized from their role during protein synthesis. However, a growing number of other, ex‐translational functions have recently been described. The most spectacular examples come from bacterial cells and include induction of the stringent control, regulation of transcription of some operons, and control of replication of ColE1‐type plasmids by uncharged tRNA 1. In yeast and mammals, in response to starvation conditions, tRNA induces phosphorylation of eukaryotic initiation factor 2 through binding to the Gcn2 kinase which modulates the transcription of amino acid biosynthesis genes and reduces total protein biosynthesis 2, 3. Moreover, during stress response, tRNA transcription is reduced, and a retrograde transport of tRNA into the nucleus is observed 4. Also aminoacylated tRNA has been shown in recent studies to serve as substrates in biochemical processes other than translation, such as cell wall formation, tagging of proteins for degradation, aminoacylation of phospholipids in the cell membrane, and antibiotic biosynthesis 5.

Recently, using high throughput sequencing, sensitive northern blot assays or computational analysis, a novel ex‐translational role of tRNA has been revealed. In response to various stress conditions, cleavage of cytosolic tRNA into stable shorter molecules has been observed, giving rise to tRNA halves (when the cleavage occurs in the anticodon loop) and tRFs (other breakage points in tRNA) (for a review, see 6 and references therein). To date, the function of tRNA‐derived fragments has been studied in many organisms and it appeared that those molecules represent a wide functional repertoire. tRNA halves seem to act as global translation regulators by displacing eIF4G/eIF4A from uncapped to capped RNA in human cell lines 7. Shorter tRFs can bind directly to 30S ribosomal subunits and inhibit translation in *Archaea* 8. tRNA fragments have also been identified in pools of small RNA copurified with Argonaute and Piwi complexes 9, 10 or being processed by Dicer 9, 11, suggesting that they could function in a way similar to siRNAs or miRNAs. Although regulation of gene expression by miRNAs is conserved in diverse eukaryotic species, including budding yeast *Saccharomyces castellii* and *Candida albicans* 12, it has been lost in *Saccharomyces cerevisiae*. Thus, this organism provides an optimal system for studying the Dicer/Argonaute‐independent mechanisms of tRNA‐derived fragments’ biogenesis and function.

It has been shown that tRNA fragments are present in small amounts even in unstressed cells, but they are highly induced when cell is exposed to stress conditions, like heat shock, low temperature, amino acid, or phosphate starvation, oxidative stress, high pH, and during development (6 and references therein). In this aspect, the best studied are tRNA halves which are generated by specific ribonucleases that are secreted from the stressed cells. These are Rny1p in yeast 13 and angiogenin in mammalian cells 14.

In 2014, the computational meta‐analysis of more than 50 short RNA libraries revealed that fragments derived from tRNA are present in all domains of life (bacteria to humans) 15. However, during estimation of their length and abundance with the use of deep‐sequencing methods, two important issues have to be addressed. First, during the cDNA library preparation, tRNA modifications can affect reverse transcriptase and therefore lead to detection of spurious truncated tRNA fragments. Since tRNA molecules are extremely abundant in cells, such truncated cDNAs could be observed in cDNA libraries. Second, cDNA libraries in the mentioned study were originally developed for detection of miRNAs, thus usually only RNA of a length less than 30 nucleotides were included (36 nucleotide of sequencing read minus significant 3′ adaptor overlap ensuring the full‐length small RNA has been sequenced). Therefore, the amounts of the tRNA fragments longer than 30 nucleotides, including tRNA halves, could be currently underestimated. On the other hand, the employment of other experimental approaches is hampered by relatively low concentration of tRNA fragments in the cells, as has been reported in previous studies 13, 16. It has been shown, however, that different purification methods may significantly affect the composition of RNA species in the isolated RNA fractions 17. Several studies have tackled this point, focusing on methods for miRNA extraction 18, 19, 20. The influence of RNA isolation on recovery of tRNA‐derived fragments which differ from miRNAs in GC content and structural features has not yet been studied.

Considering all of the above, we decided to perform the comprehensive analysis of tRNA fragment abundance in yeast *S. cerevisiae* under 12 different growth conditions. In order to measure the abundance of highly modified tRNA‐derived fragments, we have employed the northern blotting method, which is independent from the reverse transcription. In our study, we have also verified the influence of four different RNA isolation methods on tRNA fragments’ recovery, revealing that this initial step is of major impact on observed fragments’ quantities.

## Results

### RNA isolation method severely influences the detection of tRNA‐derived fragments

**Figure 1:**
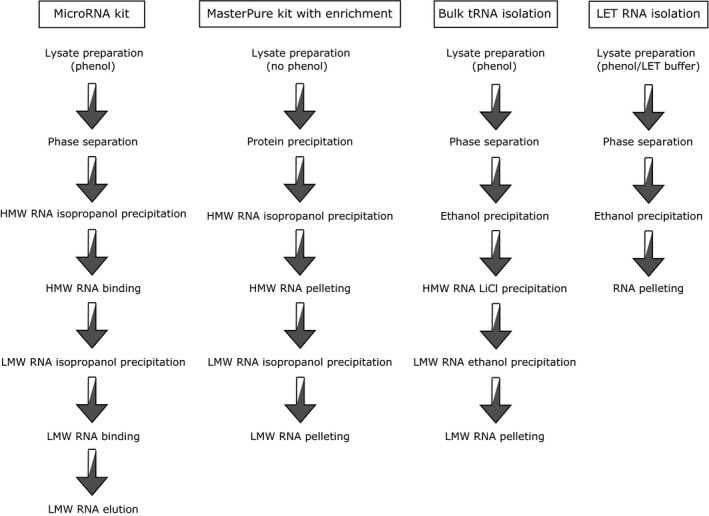
Schematic illustration of different RNA isolation methods used in this study.

At the very first step, we decided to compare the efficiency of recovery of tRNA fragments between four different methods of RNA isolation. In addition to well proven total RNA isolation method LET, which was used previously for studies on tRNA fragments in *S. cerevisiae* 13, we decided to verify the efficiency of three additional protocols resulting in enrichment of low molecular weight (LMW) RNA in the sample: standard MicroRNA kit, MasterPure kit with isopropanol enrichment step and the method widely used for isolation of bulk tRNA. The basic outline of these four methods is presented in Fig. 1.

In terms of total RNA quantity, as measured by a Nanodrop, the LET method resulted in highest yield of RNA (1.5–6‐fold higher). Among the methods for LMW RNA isolation, the median RNA yield obtained with the bulk tRNA isolation method was about 2.4‐fold higher than that obtained with MasterPure and about 1.7 higher than with MicroRNA kit. Measurement of absorbance ratio at 260 nm and 280 nm (A260/280) showed that no significant differences were observed in term of RNA purity. The variation in RNA quantities obtained from different stress conditions was in all methods about twofold.

**Figure 2:**
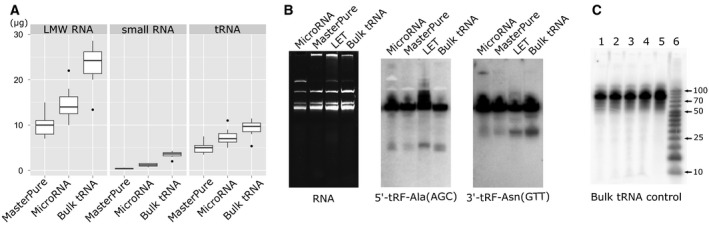
Comparison of LMW RNA extraction methods. (A) Box plot diagram showing the distributions of the absolute RNA amounts [μg] obtained from 3 × 10^7^ *S. cerevisiae* cells grown in 12 different conditions. Three different isolation methods, MasterPure, Micro RNA, and bulk tRNA are compared. Panels are representing the amounts of low molecular weight RNA (LMW, < 200 nt), small RNA (14–40 nt), and tRNA obtained with every method. The quantities were measured by Bioanalyzer 2100 using Small RNA kit. Central lines represent the medians, boxes indicate the range from 25th to 75th percentile, whiskers extend 1.5 times the above interquartile range, outliers are represented as dots. *n* = 12 sample points for all panels. (B) PAGE result showing RNA samples obtained by the employment of four different isolation procedures: Micro RNA, MasterPure, LET and bulk tRNA, and Northern blot hybridization result. Detection of 5′‐tRF‐Ala(AGC) and 3′‐tRNA‐Asn(GTT) is shown. All membranes were exposed for 16 h. Differential recovery of tRF can be observed. (C) Visualization of exogenous cellular tRNA pool (1) added at the following steps of the bulk tRNA isolation procedure: 2) directly to the cell pellet; 3) to the unbuffered phenol before shaking; 4) to the aqueous phase after phenol extraction and 5) during removal of ribosomal RNAs with LiCl. 6—size marker.

More significant differences between the tested methods were observed in recovery of short RNA fraction, which contain the tRNA‐derived fragments (14–40 nt, Fig. 2A). For those measurements, we have used the most reliable method available (Bioanalyzer 2100; Agilent Technologies, Santa Clara, CA, USA), which, however, excluded the LET‐isolated RNA pool from this comparison, due to the RNA size composition of the sample. In RNA pool isolated with MasterPure kit (Epicentre, Madison, WI, USA), similar to the total RNA quantity, the ratio of short versus LMW RNA was the lowest (median among growth conditions of 3%). In contrast, the content of short RNA obtained with MicroRNA kit (A&A Biotechnology, Gdynia, Poland) and bulk tRNA extraction methods was threefold higher with median of 9%. Interestingly, the full‐length tRNA content was similar in all isolation methods with a median amount of 50%. Overall, taking into consideration the quantity of LMW RNA as well as short RNA content, the bulk RNA isolation method resulted in 9.1‐fold higher median yields of short (14–40 nucleotides) RNA compared to the two other methods for LMW RNA isolation.

In order to provide a reliable comparison of RNA derived from all four different isolation procedures and to address the LET isolation method missing in Bioanalyzer analysis, we have additionally checked the quality of RNA (Fig. 2B). After loading 5 μg of the total LET RNA or 2.5 μg of low molecular weight RNA (derived from MicroRNA kit isolation, MasterPure kit isolation or bulk tRNA isolation) on 12% polyacrylamide gels and SYBR^®^ Safe staining, we clearly observed good separation of distinct RNA, including bulk tRNA, 5S rRNA, and a portion of small RNA. RNA isolated with the LET method were additionally enriched with the high molecular weight RNA, as expected.

In order to verify whether short RNA content in the sample is correlated with the recovery of tRNA‐derived fragments, we have performed northern blot experiments using the probe specific for the 5′ part of tRNA‐Ala(AGC) and for the 3′ part of tRNA‐Asn(GTT). As expected from our previous observations, also in tRNA fragments’ recovery, the bulk tRNA isolation method was very efficient. The MicroRNA kit and MasterPure methods failed to provide amounts of tRNA fragments above the clear detection threshold. The LET isolation method, which was previously used in yeast tRNA‐derived fragments research 13, 16 resulted in a visible detection of tRNA fragments. However, when we compared the intensity of bands corresponding to the tRF in relation to the tRNA intensity, the bulk tRNA isolation method clearly outperformed the LET isolation method (tRF signals were ~ five times more intense). The above observations indicate that compared RNA isolation methods differ not only in the amounts of short RNA but also in the composition of the short RNA fractions.

The surprisingly high performance of bulk tRNA isolation method in recovery of tRNA‐derived fragments from every growth condition raises a concern about the method reliability. It could be possible that by release or activation of cellular nucleases during the isolation procedure, tRNA could be subjected to nonphysiological degradation. In order to verify this scenario, we have spiked‐in the previously isolated and radiolabeled full‐length yeast tRNA at various steps of the bulk tRNA isolation procedure. The gel electrophoresis and autoradiography of isolated RNA did not reveal any degradation of spiked‐in tRNA, thus confirming the cellular origin of high amounts of observed tRNA‐derived fragments (Fig. 2C).

Based on above results for further investigation of tRNA‐derived fragments in yeast, we have used the bulk tRNA isolation method.

### All tested tRNA isoforms are processed into tRNA fragments

**Figure 3:**
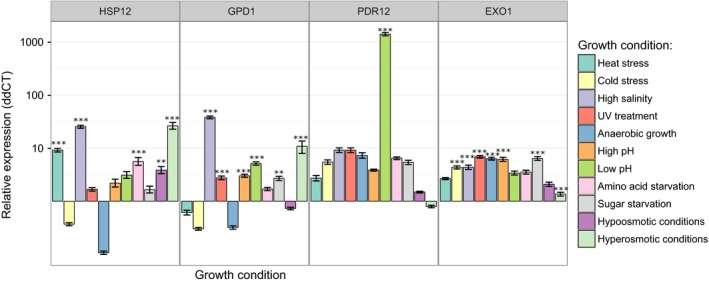
Status of a cell stress analyzed by quantitative real‐time PCR method. The expression change of four stress‐regulated yeast genes (HSP12, GPD1, PDR12, and EXO1) is shown. Expression changes are presented in log_10_ scale as the expression relative to the optimal growth conditions (∆∆CT values). Significance was designated as ** *P* ˂ 0.01, and *** *P* ˂ 0.001.

To determine the extent of tRNA isoforms that are the source of stable tRNA fragments, we performed northern blot hybridization experiments using LMW RNA pools (up to ~ 200 nucleotides) isolated with bulk tRNA method. Due to the significant changes in cell metabolism, we have used yeast cells subjected to 12 different environmental conditions: heat shock, high salinity, UV irradiation, anaerobic and optimal growth, high or low pH conditions, amino acid depletion, sugar starvation, and hypo‐ and hyper‐osmotic conditions 13, 16, 21, 22, 23. We have grown *S. cerevisiae* in optimal conditions overnight. The stress was applied for 15 min as it has been shown that rapid transcriptome changes occur most efficiently 15–20 min after stress initiation 22, 23. We have controlled the status of the cell stress by analyzing the expression of four selected well‐known stress‐regulated genes: HSP12, GPD1, PDR12, and EXO1. These genes were selected to cover all stress conditions used in this study. Full description of selected genes as well as their expression changes are summarized in Table 1. Our quantitative real‐time PCR analysis showed that the HSP12, GPD1, PDR12, and EXO1 gene expression changes under particular stresses when compared to the optimal growth conditions as expected, thus confirming the induction of the stress response in yeast cells (Fig. 3 and Table 1).

**Table 1:** Genes used in this study as markers for stress conditions

Gene name	Gene description	Expression change	
		Literature	This study
HSP12 (heat‐shock protein 12)	Plasma membrane protein involved in maintaining organization during multiple stress conditions	Upregulation during: heat shock, oxidative stress, osmostress, stationary phase, glucose depletion, oleate, alcohol 22, 23, 27, 28, 29, 30, 31	Significant upregulation during: heat shock (10‐fold), osmostress (high salt—50‐fold, and hyper‐osmotic conditions—50‐fold) Significant downregulation during cold shock (fivefold) and anaerobic growth (40‐fold)
GPD1 (NAD‐dependent glycerol‐3‐phosphate dehydrogenase)	Key enzyme of glycerol synthesis	Upregulation during: osmostress 22, 23, 28, 32	Significant upregulation during: osmostress: high salt (50‐fold) and hyper‐osmotic conditions (10‐fold)
PDR12 (plasma membrane ATP‐binding cassette ABC transporter)	Required for weak organic acid resistance	Upregulation during: low pH 22, 23, 33	Significant upregulation during low pH (1000‐fold)
EXO1 (5′‐3′ exonuclease and flap‐endonuclease)	Involved in recombination and double‐strand break repair, UV‐sensitive	Upregulation during: UV 34	The highest upregulation during UV stress (eightfold), also during anaerobic growth (sevenfold) and under high pH (sixfold)

**Figure 4:**
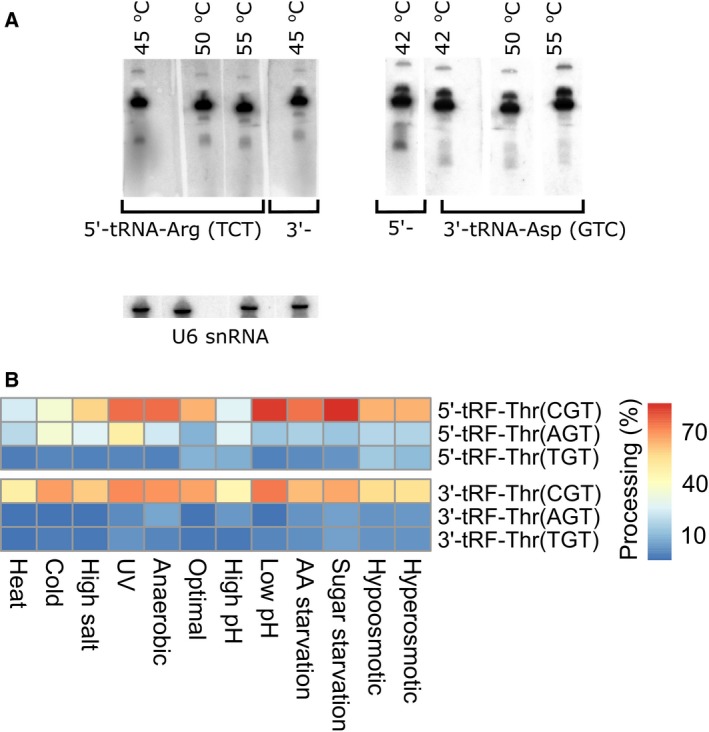
Verification of the possibility to distinguish the differential processing of tRNA isoforms. (A) Northern blot hybridization result. Detection of 5′‐ and 3′‐tRNA‐Arg (TCT) and 5′‐ and 3′‐ tRNA‐Asp (GTC) at different hybridization temperatures. U6 snRNA served as a loading control. (B) Differential processing of 3′ and 5′ parts of individual threonine tRNA isoforms. Absolute processing efficiency, calculated as the percentage of tRNA‐derived fragment signal compared to overall (fragment + tRNA) signal, is encoded in a color scale.

Although there are 41 unique mature tRNA species encoded by 275 tRNA genes in yeast (according to gtRNAdb), by careful selection of target regions, we were able to design the isoform‐specific probes that are able to detect 40 isoform variants. Due to the experimental setup, we were not able to examine the individual processing of tRNA‐Ser(TGA) and tRNA‐Ser(CGA), since the hybridization probes were identical. Therefore, both isoforms were investigated as a pool. However, we were able to design 80 specific antisense DNA probes complementary either to the 5′ or 3′ part of 40 yeast tRNA isoforms (Table S1). For some of the probes, the differences between the optimal hybridization temperature (*T*_H_) for the 3′‐part‐ and 5′‐part‐derived tRNA fragment of the same tRNA isoform reached 10–12 °C. Therefore, in order to provide a reliable comparison between different northern blot results, we have examined the possibility of differential efficiency of hybridization of antisense DNA probes to the full‐length tRNA, depending on the hybridization temperatures influencing tRNA secondary structure melting. We have selected two tRNA species for which the optimal northern blot hybridization temperature calculated by us varied most between the 5′‐part‐ and 3′‐part‐derived tRNA fragments. These were: tRNA‐Arg(TCT) (*T*_H_ for the 5′ probe: 55 °C; *T*_H_ for the 3′ probe: 45 °C) and tRNA‐Asp(GTC) (*T*_H_ for the 5′ probe: 42 °C; *T*_H_ for the 3′ probe: 55 °C). We performed the northern blot hybridization assays aiming at detection of the above tRNA at four different temperatures and compared the results with the optimal *T*_H_ (Fig. 4A). We have quantified the signal intensities of full‐length tRNA (hybridized at different temperatures) and normalized it to the signals of a loading control, U6 snRNA (hybridized at a constant temperature). In all cases, the intensity of a band corresponding to the full‐length tRNA did not change in optimal and nonoptimal temperatures. The values of normalized signal intensities were in a range from 112 073 to 113 472 for tRNA‐Arg(TCT) with a mean value of 112 845 (SD = 718) and from 125 008 to 125 845 for tRNA‐Asp(GTC) with a mean value of 125 479 (SD = 351). These results indicate that (a) different temperatures of hybridization used in this study for 5′‐part‐ and 3′‐part‐derived fragments of the same tRNA do not change the hybridization efficiency to the full‐length tRNA and (b) observed differences in tRFs abundance and processing efficiency are indeed related to their different levels. As a result, we were able to observe different patterns of tRF levels among the growth conditions for closely related probes, for example, 5′ parts and 3′ parts of three isoforms of tRNA‐Thr (Fig. 4B). However, despite the efforts to ensure the probe specificity, we do not know to what extent the probes used are complementary to the processed tRNA fragments. Thus, the probes might possibly hybridize with different efficiencies and we cannot exclude the possibility of minor cross‐hybridization.

Using the optimized RNA isolation protocol, we were able to detect fragments derived from all the 40 tested tRNA isoforms (Fig. S1). We estimated the abundance of the fragments as a percentage of the fragment's band intensity divided by the sum of fragment and full‐length tRNA band intensity (after background subtraction). In order to provide a robust quantification, the autoradiograms, revealed by quantification software to exhibit full saturation of the quantified bands, were re‐exposed. Within every group of tRNA isoforms specific for same amino acid, we have observed that at least one of the 5′ or 3′‐derived fragments was present in much higher amounts than others. The most preferentially processed tRNA isoforms include: tRNA‐Ala(TGC), tRNA‐Arg(CCT), tRNA‐Asn(GTT), tRNA‐Asp(GTC), tRNA‐Gly(CCC), tRNA‐Gly(GCC), tRNA‐Met(CAT), tRNA‐Lys(CTT), tRNA‐Leu(TAA), and tRNA‐Thr(CGT). Interestingly, in three of these cases (tRNA‐Gly, tRNA‐Lys and tRNA‐Thr), we observed isoform‐dependent accumulation of both, 5′‐ and 3′‐part‐derived fragments.

### The composition but not total amount of the tRNA‐derived fragments’ pool depends on the growth condition

**Figure 5:**
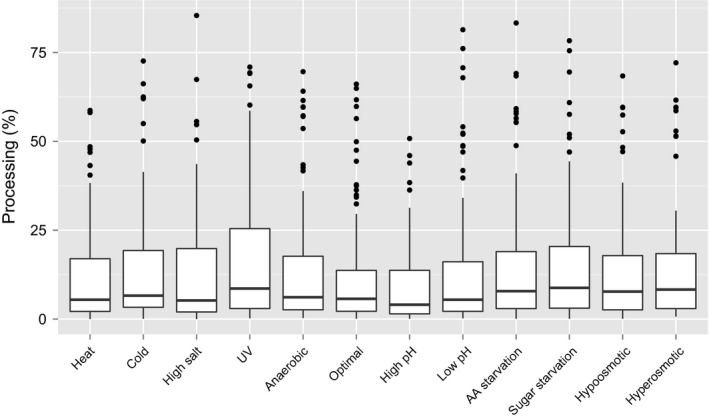
Distribution of tRNA processing efficiency in 12 growth conditions. Box plot representing distributions of the processing efficiencies of tRNAs in individual stress conditions. Central lines represent the medians, boxes indicate the range from 25th to 75th percentile, whiskers extend 1.5 times the above interquartile range, outliers are represented as dots. *n* = 96 sample points for all conditions.

**Figure 6:**
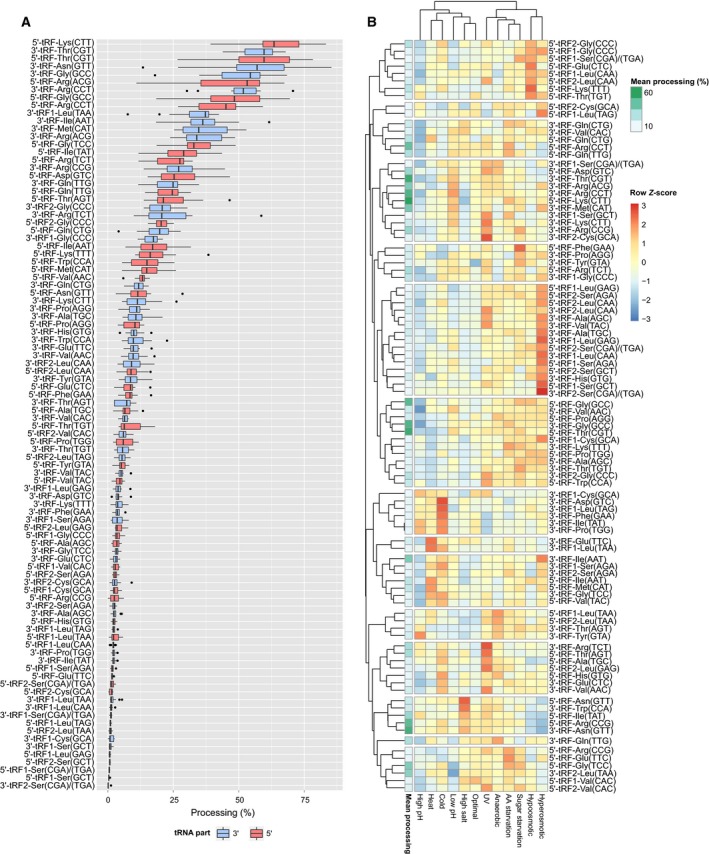
Differential processing of tRNAs. (A) Box plot representing the distributions of processing efficiencies of individual tRNA‐derived fragments among 12 different yeast growth conditions. Fragments are ordered by mean processing efficiency. tRNA fragments derived from 3′ part of tRNAs are marked with blue, and those derived from 5′ part with red. 2 tRFs, derived from the same part of particular tRNA are marked as follows: tRF1—longer tRF, tRF2—shorter tRF. Central lines represent the medians, boxes indicate the range from 25th to 75th percentile, whiskers extend 1.5 times the above interquartile range, outliers are represented as dots. *n* = 12 sample points for all tRNA fragments. (B) Clustered heat map representing the variations in tRNA processing efficiencies among 12 yeast growth conditions. The color scale encodes for the normalized *Z*‐scores calculated within the rows of the matrix, representing deviation of processing of a given tRNA fragment in a given condition from the mean processing of a given tRNA. In the first column, in a green scale, the mean processing efficiency of tRNAs has been presented.

In order to verify whether the processing of tRNA to shorter fragments is stress‐dependent, we have compared the cleavage efficiencies among 12 tested environmental conditions of *S. cerevisiae* growth. Surprisingly, we did not observe any significant differences in global efficiency of tRNA processing, neither between individual stress conditions nor in comparison to optimal growth conditions (Fig. 5). Most of the tRNA were processed with 2–20% efficiency, with the median value for each condition varying between 2–4%. However, highly abundant tRNA‐derived fragments showed preferential accumulation under high salt and low pH conditions as well as under amino acids or sugar starvation. We did not observe any highly abundant tRNA fragments under high pH conditions and just two fragments in heat stress. Next, we have investigated the changes in accumulation of individual tRNA‐derived fragments among different yeast growth conditions. The first observation was that both the median and the dynamic range of tRNA processing efficiency significantly differ between individual tRNA fragments (Fig. 6A). The lowest observed processing efficiency was 0.1% for 3′‐tRNA‐Ser(CGA) and the highest was 85.4% for 3′‐tRNA‐Asn(GTT). We have noticed that tRNA fragments which are present in high amounts are usually differentially accumulated between individual growth conditions in terms of absolute tRNA processing efficiency. However, based on the analysis of normalized changes in tRNA processing efficiency (calculation of *Z*‐scores which represent the deviation from mean processing of a given tRNA among growth conditions), we have concluded that in fact all tRNA fragments reveal similar degree of accumulation variability (Fig. 6B). Our data suggest that there are only minor differences in global tRNA cleavage efficiency between optimal and stress conditions. However, clustering of the tRNA processing profiles revealed two distinct groups of yeast growth conditions which were characterized with slightly different relative tRNA processing patterns. Under UV irradiation, anaerobic, AA starvation, sugar starvation, hypoosmotic, and hyperosmotic growth conditions, most of the tRNA fragments were accumulating more efficiently than the average, whereas in optimal, high salt, low pH, high pH, and cold and heat conditions, the relative processing seemed to be lower than the average. Those differences are only visible when analyzing the *Z*‐score‐transformed data, but not when comparing the absolute processing efficiencies (Fig. 6B versus Fig. 5). Looking at the individual tRNA fragments, we were able to observe significant differences in stress‐dependent cleavage. Interestingly, most of the tRNA fragments could be grouped into clusters of similar accumulation patterns. Those observations suggest that in general, tRNA processing in *S. cerevisiae* is not stress‐induced, but the composition of the tRNA‐derived fragments’ pool differs with changes in growth conditions.

**Figure 7:**
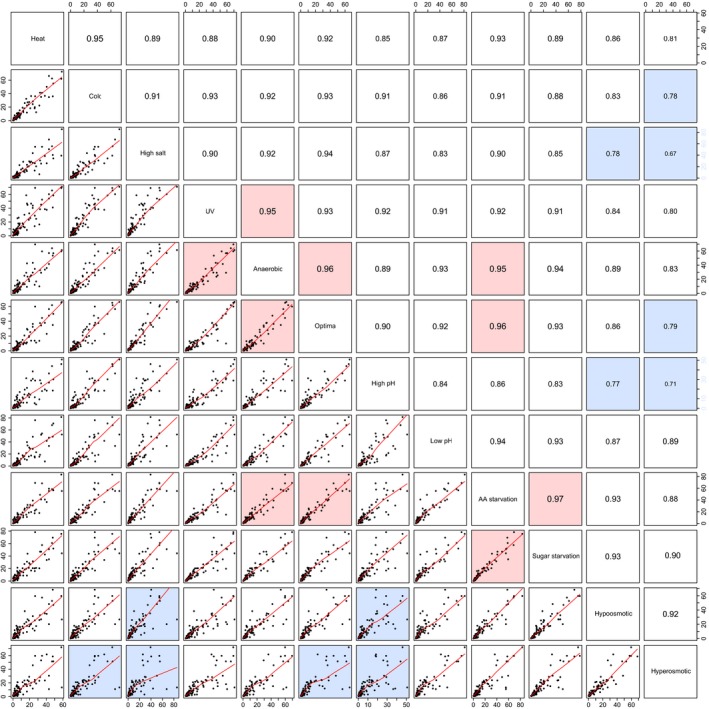
Cross‐comparison of the tRNA processing efficiencies. Scatter plot matrix containing the series of 1 : 1 comparisons of tRNA processing efficiencies between individual growth conditions. The lower triangle of the matrix represents the scatter plots of tRNA‐derived signals from compared growth conditions together with the loess fit (the red line). The upper triangle of the matrix represents the Pearson correlation of the tRNA processing between given growth conditions. Fields highlighted in red represent the conditions with highest correlation (lowest variation) of the tRNA processing, fields highlighted in blue represent the lowest correlation (highest variation). *n* = 96 for every comparison.

To investigate this hypothesis in more detail, we have analyzed the correlation between the absolute accumulation of all tRNA‐derived fragments and individual growth conditions (Fig. 7). In most of the comparisons, the Pearson correlation was in the range 0.86–0.94 (median: 0.9), suggesting that in most of the growth condition comparisons, tRNA reveal similar processing level. It was especially striking in the case of comparisons to the optimal growth conditions (median 0.93). On the other hand, the largest changes in tRF levels were observed for hyperosmotic stress: 0.81 median correlation with other growth conditions, with the lowest values for comparison with high salt conditions (0.67), high pH (0.71), cold stress (0.78), or with optimal growth conditions (0.79).

### 3′‐part‐derived tRNA fragments are as abundant as the 5′‐ ones

**Figure 8:**
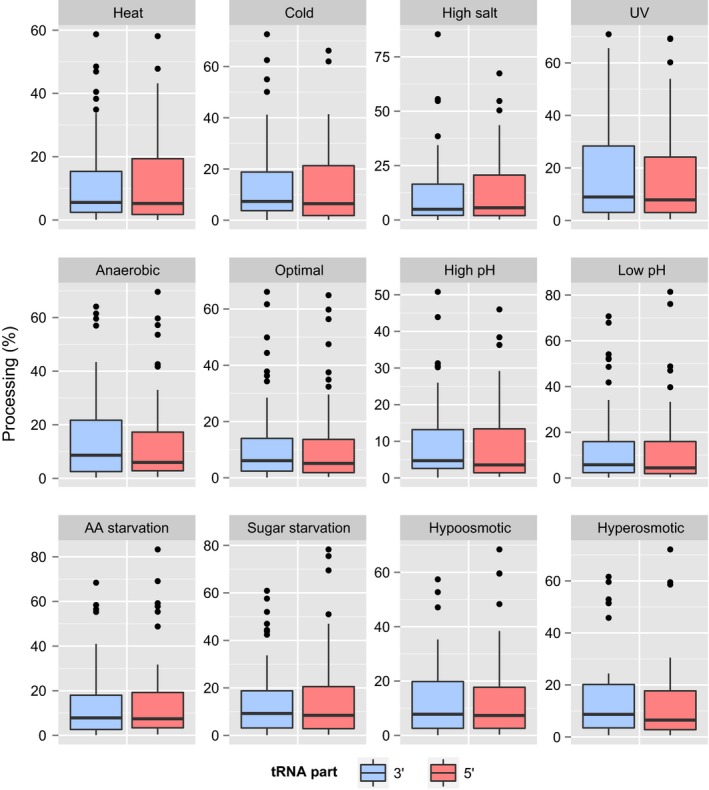
Comparison of 3′‐ and 5′‐derived tRNA fragments processing. Box plot representing the distribution of processing efficiencies of 3′‐ and 5′‐derived tRNA fragments in 12 yeast growth conditions. Central lines represent the medians, boxes indicate the range from 25th to 75th percentile, whiskers extend 1.5 times the above interquartile range, outliers are represented as dots. *n* = 46 for 3′ and 50 for 5′ fragments for all panels.

We have compared the abundance of fragments derived either from 5′ or from 3′ part of tRNA molecules. In a previous report, based on the analysis of 50 different small RNA‐seq libraries, it was shown that 5′‐tRFs were present in higher abundance than 3′‐tRFs in mouse, *Drosophila* and *Schizosaccharomyces pombe* 15. In our data, we have observed that in all tested growth conditions, both 5′‐derived as well as 3′‐derived tRNA fragments’ pools were almost equally abundant (Fig. 8). However, we observed individual differences in the levels of fragments derived either from 5′‐part or 3′‐part of the same tRNA. We found 10 most prominent examples where the abundance differences between parts of the same tRNA reached almost 50% (Fig. 6A). These were: tRNA‐Arg(CCG), tRNA‐Asn(GTT), tRNA‐Asp(GTC), tRNA‐Gly(TCC), tRNA‐Gly(CCC), tRNA‐Ile(TAT), tRNA‐Leu(TAA), tRNA‐Lys(CTT), tRNA‐Lys(TTT), and tRNA‐Thr(AGT). In six of these tRNA species, fragment derived from the 5′‐part was more stable.

## Discussion

Previous studies by Thompson *et al*. 13 demonstrated that *S. cerevisiae* contains small RNA populations consisting primarily of tRNA halves and rRNA fragments. In our recent studies, by the employment of high throughput sequencing, we have found similar tRNA‐derived RNA to interact with yeast ribosomes 21. In this study, we have performed an independent cross‐comparison of three extraction procedures for low molecular weight (LMW) RNA isolation form *S. cerevisiae* samples using MicroRNA kit, MasterPure kit with isopropanol enrichment and bulk tRNA isolation method. We have compared their efficiency in recovery of tRNA‐derived fragments with the total RNA isolation method which was previously successfully used to detect several tRNA fragments in yeast under oxidative stress, methionine starvation, nitrogen starvation, heat shock, and entry into the stationary phase 13, 16. All four methods evaluated in this study are widely applied for RNA isolation, but employ different biochemical principles. The first method, MicroRNA kit from A&A Biotechnology, employs phenol/chloroform extraction with a column‐based enrichment of RNA molecules of size below 200 nucleotides. Although in our experiments, this pool contained 6–10% of short RNA (14–40 nucleotides), we were unable to detect any clear northern blot signals corresponding to the tested tRNA fragments, while full‐length tRNA were highly abundant. We have obtained similar results for MasterPure kit with isopropanol enrichment of LMW RNA. Although this method was characterized with the lowest short RNA/LMW RNA ratio of ~ 2–4%, we were able to detect minor amounts of tRNA‐derived fragments with northern blot. The third method of LMW RNA isolation, the bulk tRNA protocol 23, gave superior results in both short RNA/LMW RNA ratio of 8–14% as well as tRNA‐derived fragments’ detection with northern blots (up to over fourfold more intensive signal for tRNA fragment than for full‐length tRNA). We compared the efficiency of all three LMW RNA isolation methods with LET protocol for total RNA extraction which previously gave positive results when detecting tRNA fragments in several stress‐derived RNA pools 13, 16. However, the intensity of the tRNA fragment‐specific northern blot signals obtained with LET method was comparable to those obtained with the MasterPure kit and clearly weaker than with the bulk tRNA isolation method.

It was surprising for us to observe the lack of correlation between the amounts of short RNA isolated with individual methods and the ability to detect tRNA‐derived fragments. This suggests that there are some specific interactions of tRNA‐derived fragments which might influence the efficiency of their extraction. One possibility that has already been shown is that some small RNA species can bind to larger RNA molecules, and therefore may comigrate with high molecular weight RNA. This results in loss of small RNA and may introduce sample–to‐sample variation in the composition and abundance of small RNA species. This phenomenon, named as the carrier effect of cellular RNA, has already been observed by Podolska *et al*. 20 in the miRVana‐isolated RNA samples tested for the presence of miRNA and further proved by Kim *et al*. 17. Another possible explanation could be that the presence of additional compounds (associated proteins and/or nucleic acids) can affect the efficiency of the tRNA fragments’ extraction. These compounds could further be lost during the RNA purification procedure. However, their presence in the initial steps of the purification could severely influence the composition of the purified RNA. This issue has already been postulated by Monleau *et al*. 18 Since we were able to gain the best results with the method that was optimized for isolation of bulk tRNA, we speculate that the mechanism of tRNA‐derived fragments loss during the isolation procedure is related to specific characteristics of tRNA molecules, including sequence, structure or high molecular interaction partners.

It was postulated by several research groups that the cleavage of tRNA is a stress‐related phenomenon. This dependence was especially well described in higher eukaryotes for tRNA halves derived by angiogenin activity 7 and tRNA fragments processed from pre‐tRNA transcripts 9. Site‐specific tRNA cleavage in *S. cerevisiae* was only observed in a limited subset of stress conditions, for example, during oxidative stress or heat shock 13, 16. The same studies in *S. cerevisiae* revealed that tRNA cleavage was not detected in yeast cells undergoing amino acid or glucose starvation and UV irradiation 16. The failure of these stress stimuli to increase tRNA fragment levels in *S. cerevisiae* suggested that the cleavage is neither a general mechanism of stress response nor a general effect of a decrease in translation rates. It has been also reported that in unstressed *S. cerevisiae* cells, only low levels a of tRNA cleavage may be detected 16. However, our data presented in this manuscript suggest that the tRNA cleavage is a general phenomenon in yeast cell, occurring independently of the growth conditions.

Due to experimental design, we were unable to estimate the exact size of the observed tRNA‐derived fragments or assign whether fragments were generated from mature tRNA or pre‐tRNA. Thus, the data presented here reflect rather general overview of tRNA processing in *S. cerevisiae*. As previously suggested by several research groups (for review see 6 and references therein), we have experimentally verified that the cleavage is not limited to specific tRNA, although the relative efficiency of cleavage can differ. Moreover, we did not observe significant differences in the accumulation of total tRNA fragments pool depending on stress condition nor increase in the relation to the optimal conditions. However, our data strongly suggest that the tRNA cleavage is regulated in a stress‐dependent manner by specific selection of tRNA species which serve as substrates for a defined processing. We believe that such widespread processing of tRNA was not observed before in *S. cerevisiae* due to two major reasons, employment of the nonoptimal RNA isolation method and/or using the high throughput sequencing data for tRNA fragments profiling, which are hampered by not well characterized dependence of reverse transcription on numerous tRNA base modifications. Previous reports showed that the 5′‐tRFs are present in higher levels than 3′‐tRFs in mouse, *Drosophila* and yeast *S. pombe* 15. However, we observed that the total pools of 5′‐derived as well as 3′‐derived tRNA fragments are almost equally abundant, independently of *S. cerevisiae* growth conditions.

In most cases reported until now, full‐length tRNA levels do not decline significantly when the tRNA processing is observed and tRNA fragments’ levels are consistently lower than those of full‐length tRNA 14, 16. This suggests that only a small portion of tRNA could be targeted as a substrate for cleavage. This is in contrast to the complete depletion of tRNA targeted by colicins [24]. This is also in contrast to our data, which show that in the case of highly and very highly abundant tRNA‐derived fragments, the signal on northern blot membrane that corresponds to the fragment is stronger than the signal of full‐length tRNA.

## Methods

### Strain and growth conditions

*Saccharomyces cerevisiae* strain BY4741 (MATα; his3Δ 1; leu2Δ 0; met15Δ 0; ura3Δ 0) was grown in synthetic optimal YPD yeast medium supplemented with 2% carbon source at 30 °C.

Cells were grown in 12 different growth conditions as described 13, 21, 22. Briefly, stress treatments were performed as follows: cells were grown to mid‐log phase (optical density at 600 nm 0.7), the stress was applied for 15 min, the cells were harvested by centrifugation and stored at −20 °C. The temperature shifts to 37 °C (heat shock) or to 15 °C (cold shock) were carried out by the addition of an equal volume of YPD prewarmed to 50 °C or chilled to 4 °C, respectively. The heat‐shocked cultures were continued to grow for 15 min at 37 °C, and cold‐shocked at 15 °C. The cultures were either supplemented with 1 m NaCl (high salt conditions), with 0.1 m Tris–HCl pH 8.3, resulting in a final pH of 7.9 (high pH conditions) or with 1 m citric acid (low pH conditions of pH 4.0). UV exposure was performed in a Stratalinker (Stratagene, La Jolla, CA, USA). Cells were grown to mid‐log phase, then moved into Petri plates and exposed to 120 J·m^−2^ UV. Yeast were returned to a flask and continued to growth for further 15 min. To induce hyperosmotic shock, the cultures were supplemented with 1 m sorbitol. For hypoosmotic conditions, the cells were grown to mid‐log phase in YPD supplemented with 1 m sorbitol, then collected by centrifugation, and resuspended in YPD without sorbitol. For amino acid and sugar starvation stresses, the cells were collected by centrifugation at mid‐log phase, washed in starvation medium and further grown in medium lacking amino acids or sugar, respectively. In parallel, anaerobic and normal growth of *S. cerevisiae* was performed.

### RNA isolation

LMW RNA (up to ~ 200 nt) were isolated from *S. cerevisiae* according to three different protocols: (a) MicroRNA isolation kit (A&A Biotechnology) following the manufacturers’ protocol, (b) MasterPure^™^ Yeast Purification kit (Epicentre) combined with the enrichment of low molecular weight RNA with isopropanol and (c) bulk tRNA isolation method as previously described 25. Additionally, total RNA was prepared as described in Thompson *et al*. 13. The same biological material derived from a single yeast culture was subjected to all three isolation procedures.

MicroRNA isolation kit requires a phenol/chloroform extraction step and purification is based on silica matrix columns. 3 × 10^7^ cells from mid‐log culture were pelleted and treated with 800 μL of Fenozol. The lysed cells were incubated at 50 °C for 5 min. 200 μL of chloroform was added and left at the room temperature for 3 min. The probes were centrifuged for 10 min at 10 000 ***g*** and nucleic acids within the resulting supernatant were precipitated with 1/3 volume of isopropanol. This mixture was filtered through the silica columns by centrifugation for 1 min at 10 000 ***g***. High molecular weight (HMW) RNA remained bound to the columns. Low molecular weight (LMW) RNA from the flow through were precipitated with 2/3 volume of isopropanol and filtered through the silica columns by centrifugation for 1 min at 10 000 ***g***. LMW RNA were recovered from the columns with 50 μL of DEPC‐treated water.

MasterPure^™^ Yeast Purification kit utilizes a simplified method for sample deproteinization: digestion of cell lysates with Proteinase K followed by a rapid desalting process to remove contaminating macromolecules 26. This method does not require a phenol/chloroform extraction step nor column‐based purifications. In this study, MasterPure kit procedure was combined with a differential isopropanol precipitation of low‐ and high molecular weight RNA; 3 × 10^7^ cells from mid‐log culture were pelleted and 300 μL of Extraction Reagent RNA containing the Proteinase K (50 μg) was added for RNA extraction and incubated at 70 °C for 15 min. The samples were then placed on ice for 5 min and 175 μL of MPC Protein Precipitation Reagent was added to the lysed sample. Cell debris were pelleted by centrifugation for 10 min at 4 °C at 10 000 ***g***. HMW RNA was precipitated with the use of 1/3 volume of the isopropanol and then discarded. LMW RNA, which remained in the supernatant were precipitated with the use of 1 volume of isopropanol.

Bulk (unfractionated) tRNA from *S. cerevisiae* were prepared as previously described 25. 3 × 10^7^ cells from mid‐log culture were pelleted and washed twice with a solution of 50 mm Na acetate pH 6.5, 10 mm MgCl_2_, and 0.1 mm EDTA. The cell pellet was immediately resuspended in 10 volumes of the same buffer (410 μL). An equal volume of unbuffered phenol 90% (equilibrated with water, Sigma) was added and mildly shook at room temperature for 15 min. Under such mild phenol treatment, mainly the ‘soluble’ RNA (essentially tRNA, 5S‐RNA and small cellular RNA) are released from the unbroken cells 23. The mix was then centrifuged for 20 min at 16 000 ***g*** and nucleic acids from the recovered aqueous phase were ethanol precipitated. The nucleic acid pellet was dissolved in 200 μL of 50 mm Na acetate, pH 6.5, 10 mm MgCl_2_, and 150 mm NaCl to which 12 m LiCl was added to reach 2 m final concentration in order to remove eventual contamination of ribosomal RNA resulting from the small fraction of cells that broke during phenol extraction (as signaled by the small amount of denatured proteins floating at the interphase of phenol:water). Following an incubation time of 1 h on ice, the insoluble ribosomal RNA was eliminated by centrifugation. Bulk ‘soluble’ RNA from supernatant (mainly tRNA, 5S RNA and small cellular RNA, as verified by electrophoresis) was recovered by ethanol precipitation.

Total RNA was prepared as previously described 13. 3 × 10^7^ cells from mid‐log culture were pelleted and resuspended in 150 μL of LET (25 mm Tris/HCl pH 8, 100 mm LiCl, 20 mm EDTA) and 150 μL of phenol equilibrated with LET. The tubes were then vortexed for 5 min with acid‐washed glass beads, after which 250 μL of phenol/chloroform equilibrated with LET and 250 μL of DEPC‐treated water were added. The aqueous phase was extracted with phenol/chloroform, chloroform extracted and ethanol precipitated.

### RNA quantity and quality

The RNA concentration and quality were first assessed using the NanoDrop ND‐1000 spectrophotometer (NanoDrop Products, Wilmington, DE, USA). The sample purity was estimated by measuring the ratio of spectrophotometric absorbance (260 nm/280 nm). For a pure RNA sample, this ratio should be comprised between 1.8 and 2. LWM RNA were further analyzed with the Bioanalyzer 2100 using Agilent Small RNA kit (Agilent Technologies).

### Quantitative reverse transcriptase PCR (qRT‐PCR) assay

The induction of stresses was verified by quantification of the expression levels of stress‐regulated genes: HSP12, GPD1, PDR12, and EXO1. Description of the genes is presented in Table 1. We have used 5S rRNA gene (RDN5) as a reference with constant expression. Reverse transcription reactions were carried out using a Superscript reverse transcriptase II (SS RT II) system (Invitrogen, Thermo Fisher Scientific, Waltham, MA, USA). Primers were used at a final concentration of 100 μm. Sequences of the primers are as follows: HSP12 Fwd 5′‐TCTTCCAAGGTGTCCACGAC‐3′; HSP12 Rev 5′‐TCGTTCAACTTGGACTTGGC‐3′; GPD1 Fwd 5′‐GGTTGGAAACATGTGGCTCT‐3′; GPD1 Rev 5′‐GGCAGGTTCTTCATTGGGTA‐3′; PDR12 Fwd 5′‐GTCGTTGAATCTGGTGAAATG‐3′; PDR12 Rev 5′‐AGACATCATTTCGCTTTGGTC‐3′; EXO1 Fwd 5′‐TGGTGATGCCATTCCAGTTA‐3′; EXO1 Rev 5′‐AACGGAGCCACTATGTACCG‐3′; RDN5 Fwd 5′‐AGATTGCAGCACCTGAGTTT3′; RDN5 Rev 5′‐GGTTGCGGCCATATCTACCA‐3′. Quantitative PCRs (25 μL) were performed on aliquots of a reverse transcription reaction using Eva green system (Solis Biodyne, Tartu, Estonia). Datasets were collected on an Agilent real‐time PCR system and analyzed using maxpro version 3.1 software (Honeywell, Louisville, KY, USA). The cycling conditions were as follows: 3 min at 95 °C, followed by 40 cycles consisting of 45 s at 94 °C, 30 s at 57 °C, and 20 s at 72 °C. Fluorescence signal data were collected during the 72 °C phase of each cycle. Melt curves from 56 °C to 95 °C (in 0.5 °C increments, measuring fluorescence at each temperature) were collected for all samples following the last cycle and showed the presence of only one product in each reaction. The standard curves were used to derive the copy number of each transcript in each RNA sample, which was determined in triplicate. Statistical analysis was performed using GraphPad Prism v5.01. Data were collected as triplicate from at least three independent experiments. The results were expressed as mean ± standard deviation (SD). Differences between the means of treatments were evaluated using one‐way analysis of variance (ANOVA) followed by Tukey's test.

### Specificity of the bulk tRNA isolation method

Cellular tRNA pool from *S. cerevisiae* was prepared with phenol/chloroform extraction method, purified with polyacrylamide gel electrophoresis and 5‐[^32^P]‐end‐labeled as previously described 21. 20 000 cpm of the tRNA pool was added during the following steps of the bulk tRNA isolation procedure: (a) directly to the cell pellet; (b) to the unbuffered phenol before shaking; (c) to the aqueous phase after phenol extraction and (d) during removal of ribosomal RNA with LiCl. In each case bulk tRNA procedure was completed according to the protocol. The resulting RNA was recovered by ethanol precipitation and separated with the use of polyacrylamide gel electrophoresis. The gels were exposed on the phosphor – storage intensity screen (Fujifilm, Tokyo, Japan) overnight. Screens were scanned with Fujifilm Fluorescent Image Analyzer FLA – 5100 and analyzed quantitatively with the densitometric program multi gauge image analyzer (Fujifilm).

### Northern blot analysis

Twenty‐five micrograms of LMW RNA or 50 μg of total RNA were separated on 12% denaturing polyacrylamide gels and electro transferred to the positively charged Amersham Hybond N^+^ membrane using a semidry blotter (BioRad) for 45 min with 0.8 mA·cm^−2^ of the membrane. Nucleic acids were UV‐cross‐linked to the membranes, which were then used immediately for northern blot hybridization or stored at room temperature. tRNA fragments were detected with antisense 5‐[^32^P]‐end‐labeled DNA probes as previously described 21. DNA oligonucleotide probes were synthesized by Genomed. The sequences as well as the hybridization temperature are presented in Table S1. Hybridization was carried out overnight in 30 mL of a buffer (178 mm Na_2_HPO_4_, 882 mm NaH_2_PO4, 7% SDS) at specific hybridization temperature (*T*_H_ = *T*_m_ – 10–15 °C) with gentle rotation. Two‐step washing was performed after hybridization at *T*_H_ with rapid rotation: for 2 min in a washing solution I (2× SSC, 0.1% SDS) and for 1 min in a washing solution II (0.1× SSC, 0.1% SDS). The membranes were exposed on the phosphor – storage intensity screen (Fujifilm) overnight. Screens were scanned with Fujifilm Fluorescent Image Analyzer FLA – 5100 and analyzed quantitatively with the densitometric program multi gauge image analyzer.

The membranes were reused for up to five times for blotting with different probes. For that reason, the detachment of radioactive probes was performed. Membranes were placed in hybridization tubes and the buffer was added (0.2× SSC, 0.5% SDS). The procedure was performed for 30 min at 85 °C with rapid rotation.

Hybridizations of every DNA probe were repeated at least twice, but in most cases, three times to different membranes, which were derived from biological replicates. Since the measurements of repetitions were highly consistent (median of relative standard deviation of quantifications = 0.158%), we decided to use the mean values for subsequent analysis. Statistical analysis of the results has been performed using R statistical environment.

## Author contributions

KBŻ planned experiments. KBŻ, AMM, MK and PM performed experiments. KBŻ, MŻ and TT analyzed data. KBŻ and MŻ wrote the paper.

## Supporting information


**Table S1.** Sequences of antisense DNA probes used in this study. Hybridization temperature is indicated (*T*_H_).
**Fig. S1.** tRNA‐derived fragments in *Saccharomyces cerevisiae*.
