# Supplementary material for: The widespread occurrence of tRNA‐derived fragments in Saccharomyces cerevisiae
Source: FEBS Open Bio. 2016 Oct 21;6(12):1186–200. doi: 10.1002/2211-5463.12127 (PMC5302060; doi:10.1002/2211-5463.12127)
Supplement: Supplementary file 1 — Table S1. Sequences of antisense DNA probes used in this study. Hybridization temperature is indicated (T H). Fig. S1. tRNA‐derived fragments in Saccharomyces cerevisiae. [file FEB4-6-1186-s001.pdf]

Supporting material

The widespread occurrence of tRNA-derived fragments in *Saccharomyces cerevisiae*

Kamilla Bąkowska-Żywicka[1*], Anna M. Mleczko[1], Marta Kasprzyk[1], Piotr Machtel[1], Marek Żywicki[2], and Tomasz Twardowski[1]

[1]Institute of Bioorganic Chemistry Polish Academy of Sciences, Noskowskiego 12/14, 61-704 Poznan, Poland

[2] Department of Computational Biology, Institute of Molecular Biology and Biotechnology, Faculty of Biology A. Mickiewicz University in Poznan, Umultowska 89, 61-614 Poznan, Poland

* To whom correspondence should be addressed. Tel: +48/618528503; Fax: +48/6185280532; Email: bakowska@ibch.poznan.pl

Table S1. Sequences of antisense DNA probes used in this study. Hybridization temperature is indicated (T_H).

tRNA isotype	5'-probe sequence	T_H	3'-probe sequence	T_H
$tRNA^{Ala}$ (AGC)	AGGGAGCGCGCTACCGACTACGCCACACGCCC	55ºC	TTGGACGAGTCCGGAATCGAACCGGAGACC	55ºC
$tRNA^{Ala}$ (TGC)	GGGAAGCGCGCTACCAACTGCGCCATGTGCCC	45ºC	TTGGACGCAACCGGAATCGAACCGATGACC	45ºC
$tRNA^{Arg}$ (ACG)	TGACCATTGGGCCACGAGGA	45ºC	CCCCGCCAGGACTCGAACCTGGAATC	55ºC
$tRNA^{Arg}$ (CCG)	GAATGCATGCTAACCATTGCACTAGAGGAGC	50ºC	TCCTCCCGGGACTCGAACCCGGATC	55ºC
$tRNA^{Arg}$ (CCT)	GGGAGACGCGTTACCATTACGCCAACGGAAC	55ºC	TCCGTACGGGACTCGAACCCGCAGT	50ºC
$tRNA^{Arg}$ (TCT)	GTCAGACGCGTTGCCATTACGCCACGCGAGC	55ºC	CGATGGGGGTCGAACCCATAATCTT	45ºC
$tRNA^{Asn}$ (GTT)	GCACGCCTTAACCAACTTGGCCATGGAGTC	50ºC	CGACCCCAGTGAGGGTTGAACTCACGATCTTGCGAT	45ºC
$tRNA^{Asp}$ (GTC)	GCGCCCATTCTGACCATTAAAC	42ºC	GGCTCCGCGACGGGGAATTGAACCCCG	55ºC
$tRNA^{Cys}$ (GCA)	GCGCTACCACTGCGCCATACGAGC	50ºC	CGCACTCAGGATCGAACTAAGGACC	50ºC
$tRNA^{Gln}$ (CTG)	CCGAAAGTGATAACCACTACACTATAGGAC	50ºC	TCCCACCCGGATTCGAACTGGGG	50ºC
$tRNA^{Gln}$ (TTG)	CCGAAAGTGATAACCACTACACTATAAAAC	42ºC	TCTTACCCGGATTCGAACCGGGG	42ºC
$tRNA^{Glu}$ (CTC)	CGTGATGTGATAGCCGTTACACTACATCGG	50ºC	GGCTCCGAAGCGGGGAGTCGAACCCCGG	55ºC
$tRNA^{Glu}$ (TTC)	CGTGATGTGATAGCCGTTACACTATATCGG	50ºC	GGCTCCGCTACGGGGAGTCGAACCCCGG	55ºC
$tRNA^{Gly}$ (CCC)	AACCACTGAACCACTTTCGC	42ºC	GAAGCCGGGAATCGAAC	42ºC
$tRNA^{Gly}$ (GCC)	TACCACTAAACCACTTGCGC	42ºC	CAAGCCCGGAATCGAAC	42ºC
$tRNA^{Gly}$ (TCC)	AACCACTACACTAACCGCCC	42ºC	GAGCGGTACGAGAATCGAAC	42ºC
$tRNA^{His}$ (GTG)	TGTGTACTAACCACTATACTAAGATGG	45ºC	AGAATCGAACCAGGGTTTCA	60ºC
$tRNA^{Ile}$ (AAT)	GCCTTAACCAACTGGGCCAAGAGACC	50ºC	TGGTCTCTAGCGGGATCG	42ºC
$tRNA^{Ile}$ (TAT)	CGAAGCTCTAACCACTGAGCTACACGAGC	50ºC	ATTGAACCCACGACGGTCGCGT	45ºC
$tRNA^{Leu}$ (CAA)	TGAATCAGGCGCCTTAGACCGCTCGGCC	55ºC	GGTTGCTAAGAGATTCGAACTCTTGCATCT	50ºC
$tRNA^{Leu}$ (GAG)	TCGACCTGACGCCTTAGACCACTCGGCC	55ºC	GATACCCGCGGGGTTTGAACCCGCGCCTCC	55ºC
$tRNA^{Leu}$ (TAA)	TAAGTCTGCCGCCTTAGACCACTCGGCC	55ºC	GAAGGATGCGAGGTTCGAACTCGCGCGGAC	42ºC
$tRNA^{Leu}$ (TAG)	TAAATCTGACGCCTTAAACCACTCGGCC	50ºC	GAGAGCTAAGGGATTCGAACCCTTGCATCC	50ºC
$tRNA^{Lys}$ (CTT)	CGCGCTACCGATTGCGCCAACAAGGC	55ºC	GGCTCGAACCCCTAACCTT	42ºC
$tRNA^{Lys}$ (TTT)	GCCGAACGCTCTACCAACTGAGC	45ºC	CTCCTCATAGGGGGCTCGAACCCC	50ºC
$tRNA^{Met}$ (CAT)	CTGACGCTCTTCCTACTGAGCTACTGAAGC	50ºC	TGCTCCAGGGGAGGTTCGAACTCTCGACCTTCAG	55ºC
$tRNA^{Phe}$ (GAA)	CTCTCCCAACTGAGCTAAATCCG	45ºC	TGCGAATTCTGTGGATCGAACACAGGACC	50ºC
$tRNA^{Pro}$ (AGG)	CGAGAATCATACCTCTAGACCACACGCCC	50ºC	CGAGCCGGGACTCGAACCCGGG	55ºC
$tRNA^{Pro}$ (TGG)	CGAGAATCATACCACTAGACCACACGCCC	50ºC	CGAGCTGGGAATTGAACCCAGG	45ºC
$tRNA^{Ser}$ (AGA)	CGCCTTAACCACTCGGCCAAGTTGCC	50ºC	ACAACTGCAGGACTCGAACC	42ºC
$tRNA^{Ser}$ (CGA)	CGCCTTAACCACTCGGCCATAGTGCC	50ºC	CGACACCAGCAGGATTTGAACCAGCG	50ºC
$tRNA^{Ser}$ (GCT)	CGCCTTAACCACTCGGCCACTGGGAC	55ºC	CGTCACAGACAGGATTCGAACCTGCG	50ºC
$tRNA^{Thr}$ (AGT)	GCCTTACCAACTTGGCCATA	42ºC	TTGAACCGATGATCTCCACA	42ºC
$tRNA^{Thr}$ (CGT)	AGTGCGATGCCTTACCACTTGGCC	45ºC	CTGTGGGAATTGAACCCACGATCCCCGC	55ºC
$tRNA^{Thr}$ (TGT)	ACAAGTGCAACGCTCTACCA	42ºC	GAATTGAACTAACGACCTTTGC	42ºC
$tRNA^{Trp}$ (CCA)	GCTCTACCATTGAGCCACCGCTTC	50ºC	TGAAACGGACAGGAATTGAACC	42ºC
$tRNA^{Tyr}$ (GTA)	GCGCCTTAAACCAACTTGGCTACCGAGAG	50ºC	TCTCCCGGGGGCGAGTCG	45ºC
$tRNA^{Val}$ (AAC)	GCCATAACCGACTAGACCACGAAAC	50ºC	ATTTCGCCCAGGAACGAACTGGGGACG	42ºC
$tRNA^{Val}$ (CAC)	CGTGATAGCCGCTACACTATTGGAAC	50ºC	GTTCCAACCGAGGATCGAACTCGGGACC	50ºC
$tRNA^{Val}$ (TAC)	CTTGAACCACTGGACCATTGGACC	50ºC	GATCCAACCGAGGTTCGAACTCGGGATC	50ºC

Figure S1. tRNA-derived fragments in *Saccharomyces cerevisiae*.

Detection of 3'- and 5'-derived tRNA fragments visualized by northern blot hybridization assays. Numbers correspond to the RNA samples derived from *S. cerevisiae* grown under the following conditions: 1- heat stress, 2 – cold stress, 3 – high salinity, 4 – UV treatment, 5 – anaerobic growth, 6 – optimal conditions, 7 – high pH, 8 – low pH, 9 – amino acid starvation, 10 – sugar starvation, 11- hypoosmotic conditions, 12 – hyperosmotic conditions.

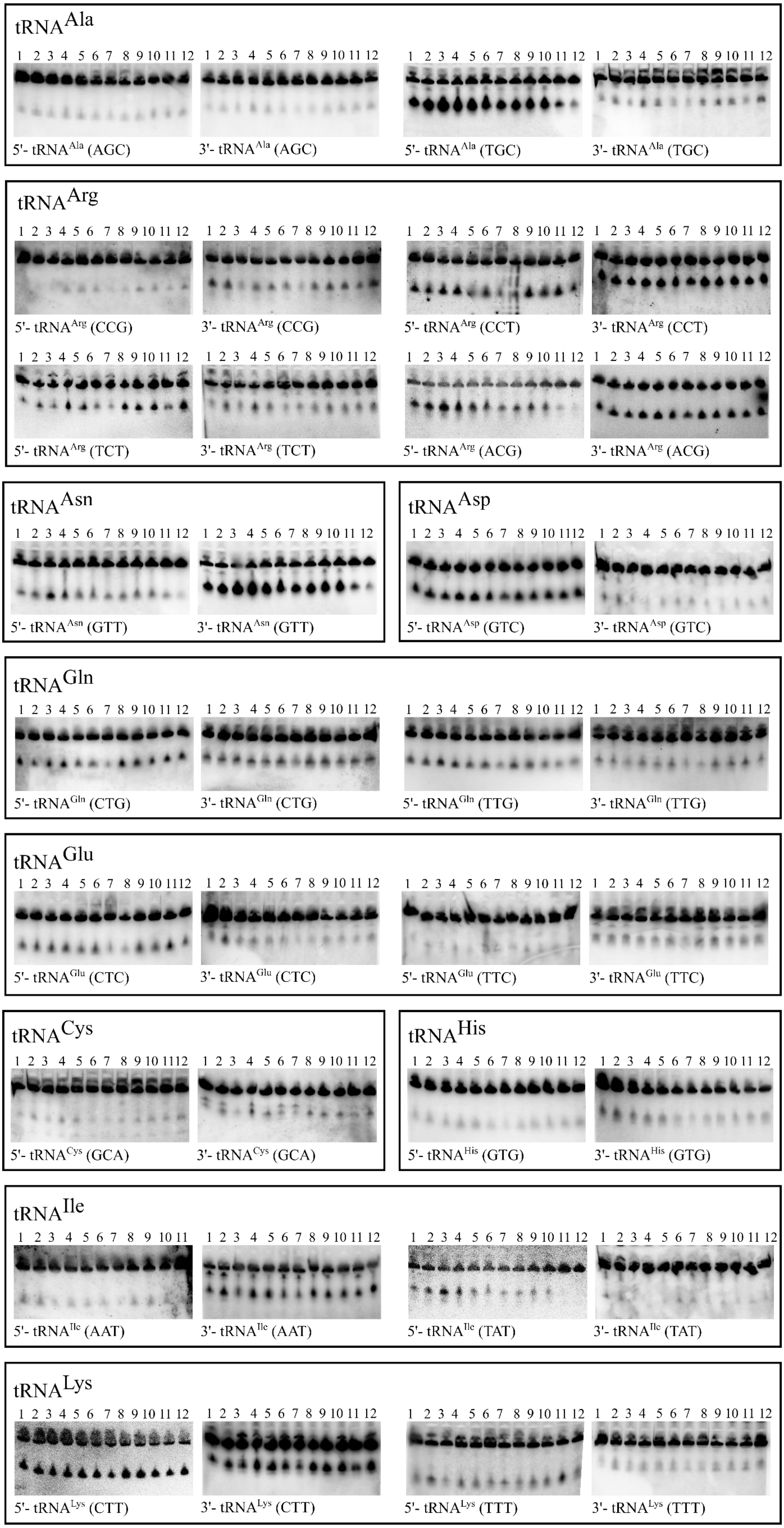
tRNA^{Ala}
5'- tRNA^{Ala} (AGC)
3'- tRNA^{Ala} (AGC)
5'- tRNA^{Ala} (TGC)
3'- tRNA^{Ala} (TGC)
tRNA^{Arg}
5'- tRNA^{Arg} (CCG)
3'- tRNA^{Arg} (CCG)
5'- tRNA^{Arg} (CCT)
3'- tRNA^{Arg} (CCT)
5'- tRNA^{Arg} (TCT)
3'- tRNA^{Arg} (TCT)
5'- tRNA^{Arg} (ACG)
3'- tRNA^{Arg} (ACG)
tRNA^{Asn}
5'- tRNA^{Asn} (GTT)
3'- tRNA^{Asn} (GTT)
tRNA^{Asp}
5'- tRNA^{Asp} (GTC)
3'- tRNA^{Asp} (GTC)
tRNA^{Gln}
5'- tRNA^{Gln} (CTG)
3'- tRNA^{Gln} (CTG)
5'- tRNA^{Gln} (TTG)
3'- tRNA^{Gln} (TTG)
tRNA^{Glu}
5'- tRNA^{Glu} (CTC)
3'- tRNA^{Glu} (CTC)
5'- tRNA^{Glu} (TTC)
3'- tRNA^{Glu} (TTC)
tRNA^{Cys}
5'- tRNA^{Cys} (GCA)
3'- tRNA^{Cys} (GCA)
tRNA^{His}
5'- tRNA^{His} (GTG)
3'- tRNA^{His} (GTG)
tRNA^{Ile}
5'- tRNA^{Ile} (AAT)
3'- tRNA^{Ile} (AAT)
5'- tRNA^{Ile} (TAT)
3'- tRNA^{Ile} (TAT)
tRNA^{Lys}
5'- tRNA^{Lys} (CTT)
3'- tRNA^{Lys} (CTT)
5'- tRNA^{Lys} (TTT)
3'- tRNA^{Lys} (TTT)

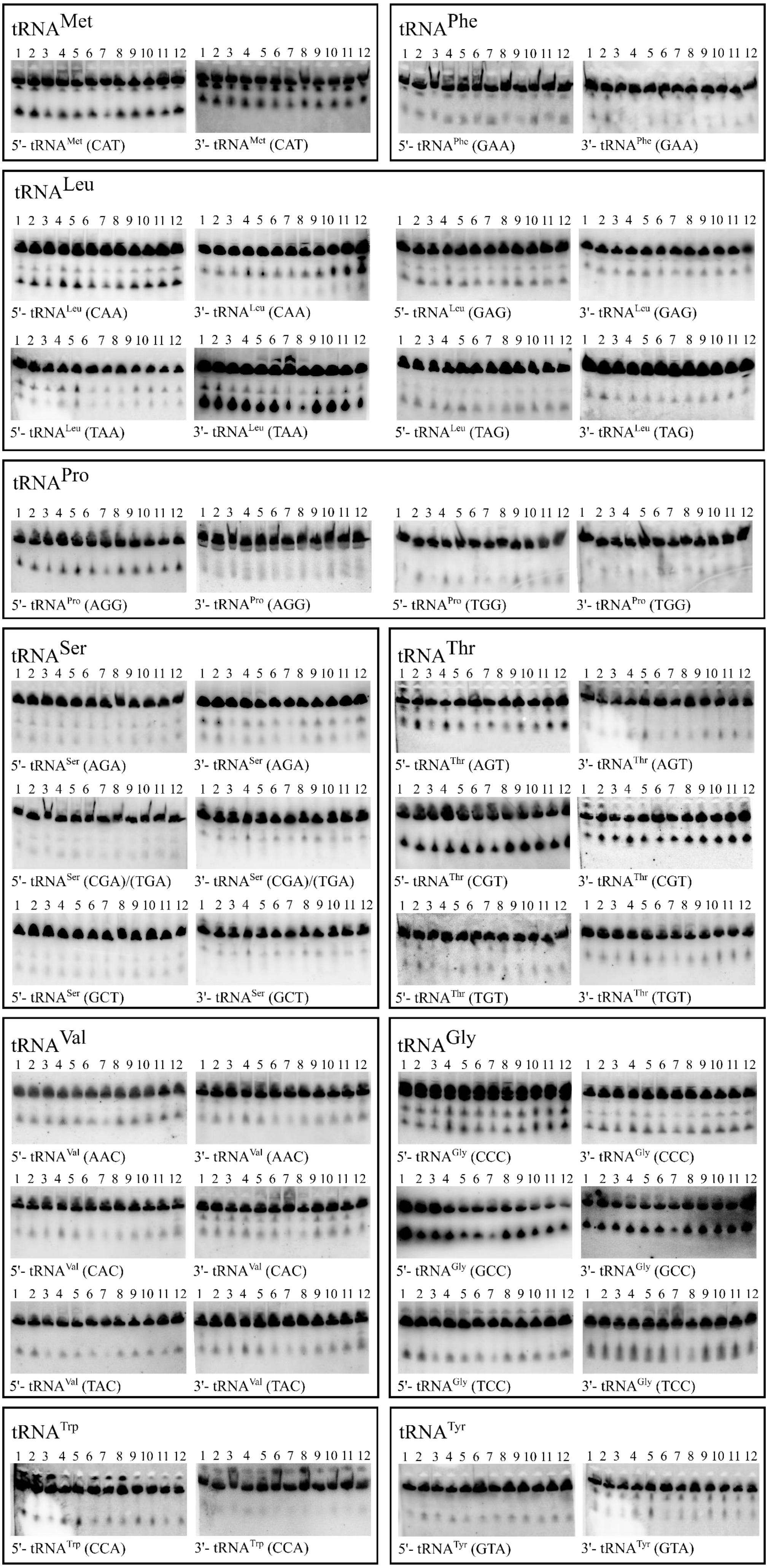

tRNA^{Met}
1 2 3 4 5 6 7 8 9 10 11 12
5'- tRNA^{Met} (CAT)
3'- tRNA^{Met} (CAT)
tRNA^{Phe}
5'- tRNA^{Phe} (GAA)
3'- tRNA^{Phe} (GAA)
tRNA^{Leu}
5'- tRNA^{Leu} (CAA)
3'- tRNA^{Leu} (CAA)
5'- tRNA^{Leu} (GAG)
3'- tRNA^{Leu} (GAG)
5'- tRNA^{Leu} (TAA)
3'- tRNA^{Leu} (TAA)
5'- tRNA^{Leu} (TAG)
3'- tRNA^{Leu} (TAG)
tRNA^{Pro}
5'- tRNA^{Pro} (AGG)
3'- tRNA^{Pro} (AGG)
5'- tRNA^{Pro} (TGG)
3'- tRNA^{Pro} (TGG)
tRNA^{Ser}
5'- tRNA^{Ser} (AGA)
3'- tRNA^{Ser} (AGA)
5'- tRNA^{Ser} (CGA)/(TGA)
3'- tRNA^{Ser} (CGA)/(TGA)
5'- tRNA^{Ser} (GCT)
3'- tRNA^{Ser} (GCT)
tRNA^{Thr}
5'- tRNA^{Thr} (AGT)
3'- tRNA^{Thr} (AGT)
5'- tRNA^{Thr} (CGT)
3'- tRNA^{Thr} (CGT)
5'- tRNA^{Thr} (TGT)
3'- tRNA^{Thr} (TGT)
tRNA^{Val}
5'- tRNA^{Val} (AAC)
3'- tRNA^{Val} (AAC)
5'- tRNA^{Val} (CAC)
3'- tRNA^{Val} (CAC)
5'- tRNA^{Val} (TAC)
3'- tRNA^{Val} (TAC)
tRNA^{Gly}
5'- tRNA^{Gly} (CCC)
3'- tRNA^{Gly} (CCC)
5'- tRNA^{Gly} (GCC)
3'- tRNA^{Gly} (GCC)
5'- tRNA^{Gly} (TCC)
3'- tRNA^{Gly} (TCC)
tRNA^{Trp}
5'- tRNA^{Trp} (CCA)
3'- tRNA^{Trp} (CCA)
tRNA^{Tyr}
5'- tRNA^{Tyr} (GTA)
3'- tRNA^{Tyr} (GTA)
